# Granulocyte-Macrophage Colony Stimulating Factor in Single Blastocyst Conditioned Medium as a Biomarker for Predicting Implantation Outcome of Embryo

**DOI:** 10.3389/fimmu.2021.679839

**Published:** 2021-06-30

**Authors:** Peilin Chen, Chunyu Huang, Qing Sun, Huixian Zhong, Feng Xiong, Su Liu, Zhihong Yao, Zhiqiang Liu, Caiyun Wan, Yong Zeng, Lianghui Diao

**Affiliations:** ^1^ Shenzhen Key Laboratory of Reproductive Immunology for Peri-Implantation, Shenzhen Zhongshan Institute for Reproduction and Genetics, Fertility Center, Shenzhen Zhongshan Urology Hospital, Shenzhen, China; ^2^ Department of Paediatrics and Adolescent Medicine, Li Ka Shing Faculty of Medicine, The University of Hong Kong, Hong Kong, China

**Keywords:** embryo quality, granulocyte-macrophage colony-stimulating factor, pregnancy outcome, single-blastocyst conditioned medium, trace protein detection

## Abstract

**Background:**

It is highly desirable to develop new strategies based on secretomics to more accurately selection of embryos with the highest developmental potential for transfer. Granulocyte-macrophage colony-stimulating factor (GM-CSF) has been reported to promote embryo development and pregnancy establishment. However, the predictive value of GM-CSF in single blastocyst selection remains unclear. This study is to determine the concentration of GM-CSF in human single-blastocyst conditioned medium (SBCM) and to evaluate its association with embryo quality and pregnancy outcome.

**Methods:**

The patients with ≤38 years of age receiving the first cycle of assisted reproductive therapy were included in this study. The patients who had <4 top-quality embryos formed by the fertilized two pronuclear zygotes on day 3 were excluded. A total of 126 SBCM samples (SBCMs) were included, of which blastocysts from 77 SBCMs were later transferred in subsequent frozen-thawed embryo transfer. The concentrations of GM-CSF were detected by single-molecule array (SIMOA) and analyzed for their possible association with embryo quality and pregnancy outcomes. The top-quality embryo (TQ), positive HCG (HP), clinical pregnancy (CP), and ongoing pregnancy (OP) rates were determined and compared between groups divided based on GM-CSF concentrations.

**Results:**

The detection rate of GM-CSF was found to be 50% in all SBCMs. There were significant differences in TQ rate, HP rate, CP rate and OP rate among high concentration group, medium concentration group and low concentration group. Both GM-CSF alone or GM-CSF combined with the morphological score (MS) had a greater AUC of ROC curve than that of MS alone to predict the pregnancy outcome, and GM-CSF combined with MS had the highest AUC.

**Conclusions:**

The concentration of GM-CSF in SBCM was detected at fg/ml levels, which was associated with embryo quality and pregnancy outcome. Collectively, GM-CSF may be used as a biomarker for prediction of pregnancy outcome and selection of embryos with high developmental potential for transfer in assisted reproductive technology (ART).

## Introduction

The primary goals of assisted reproductive technology (ART) are to perform the single-embryo transfer to achieve high live-birth rates, to minimize the chances of multiple pregnancies, and to attain higher overall successful pregnancy rates. Thus, the selection of the most viable embryos with the best developmental potential for transfer represents a vital integral part of ART (1). Conventionally, embryos are selected for transfer based on the noninvasive morphological evaluation; however, these methods are relatively subjective, and there are limitations to the predictive power of this method (2, 3). To compensate for these limitations, multiple embryos are transferred to obtain a high successful pregnancy rate, however, this leads to an increased rate of multiple pregnancies (4). Therefore, it is highly desirable to develop new strategies to accurately select embryos with the highest developmental potential for transfer.

Accumulating studies have suggested that cross-talk between embryo and endometrium, which is mediated by soluble protein and corresponding receptors, is important for embryo growth and implantation (5). Thus, new methods based on determining the proteins secreted by embryo in the culture medium have emerged to assess embryo viability and improve successful pregnancy rate (6). Granulocyte-macrophage colony-stimulating factor (GM-CSF) is a hematopoietic cytokine with multiple effects such as proliferation, differentiation and adhesion induction (7). GM-CSF in the female reproductive tract (8, 9) promotes embryo implantation and pregnancy by regulating the uterine leukocyte population (10). It was reported that preimplantation embryo could secrete GM-CSF (11). In addition, GM-CSF concentration in the culture medium was demonstrated to be associated with pregnancy outcome (12). However, due to the limited sensitivity of available testing methods, a mixture of several culture media from single-blastocyst was used to determine the concentration of GM-CSF and its relationship with pregnancy outcome. The quantitative detection of GM-CSF in human single-blastocyst conditioned medium (SBCM) has not been established. Furthermore, the association between GM-CSF concentration in SBCM with the quality of embryo and pregnancy outcome remains to be elucidated.

Single-molecule array (SIMOA) is a protein detection method based on digital enzyme-linked immunity (digital ELISA), by isolating and detecting single immunocomplexes in arrays of femtoliter-volume wells. SIMOA enables clinically important proteins to be measured at femtogram (fg/ml) concentrations (13). It has been applied in several therapeutic areas, including oncology, neurology, and immunology (14, 15). In the present study, using SIMOA, we measured GM-CSF concentration in SBCM and evaluated the association of GM-CSF with the embryo quality and pregnancy outcome.

## Materials and Methods

### Study Design

Infertile couples, who underwent IVF or ICSI cycles at the Fertility Center, Shenzhen Zhongshan Urology Hospital between March 2019 and March 2020, were retrospectively enrolled into this study. The inclusion criteria include: 1). females age less than 38 years; 2). undergoing the first cycle of assisted reproductive therapy; 3). the number of top-quality embryos, formed by the fertilized two pronuclear zygotes (2PN), was no less than four on day 3. The exclusion criteria include: 1) abnormal ultrasonography and hysterosalpingogram/hysteroscopy result. The SBCMs of the patients enrolled in this study was collected to do GM-CSF concentration assay. At last, a total of 126 SBCMs from 100 infertile couples were collected, and then all these blastocysts were frozen. Of which, 77 were subjected to embryo transfer operation and the pregnancy outcomes were followed up. For the freeze-thaw single embryo transplantation cycles, endometrium was prepared by either natural protocols or artificial protocols. For natural protocols, embryo transfer carried out on day 5 after ovulation if the endometrial thickness exceeded 7 mm. For artificial protocols, patients received 4, 6 and 8 mg oral oestradiol per day successively. When the thickness of endometrium reached 7–8 mm, progesterone was started and embryo transfer was performed on day 6 after progesterone injection. Luteal phase support was achieved until a pregnancy test was positive and was continued until 3 months of gestation. Maternal serum HCG was measured on 11 days after the embryo transfer and the HCG level higher than 5 IU/L indicated HCG positive (HP). Intrauterine gestational sac detected by transvaginal ultrasound on 30 days after the embryo transfer was considered as a clinical pregnancy (CP). Pregnancy that proceeded beyond 3 months was defined as ongoing pregnancy (OP). This study was approved by the Research Ethics Committee of Shenzhen Zhongshan Urology Hospital (Approval number: SZZSECHU-20180021).

### Human Embryo Culture

Oocytes were retrieved by an ultrasound-guided method at 36 h after administering human chorionic gonadotropin, and then IVF or ICSI was performed. Fertilization was assessed at 17 ± 1 h after insemination. The fertilized zygotes were cultured in Quinn’s Advantage Cleavage Medium (SAGE BioPharma, Bedminster, NJ, USA) supplemented with 10% (v/v) serum protein substitute (SAGE BioPharma). The cleaving embryos were evaluated on D3 according to the following criteria: the number of blastomeres, the degree of fragmentation, and the symmetry of the blastomere. In our center, a cleavage stage embryo was defined as a top-quality embryo if it meets one of the following two criteria: 1. the number of blastomeres ≥7 and ≤10 with the degree of fragmentation of <20%; 2. embryos with six symmetrical blastomeres and a degree of fragmentation of <10%. The cleaving embryo was subsequently transferred to the Quinn’s Advantage Blastocyst Medium (SAGE BioPharma) containing 10% (v/v) serum protein substitute and independently cultured until embryos reached the blastocyst stage. The morphological assessment of blastocyst was assessed on D5. The morphological assessment criteria of blastocysts were based on the Gardner system, as described previously (16). Briefly, the scores evaluate the degree of blastocyst expansion (1–6; as the embryo expands, the degree of expansion increases), the consistency of the inner cell mass (A–C; A being the highest), and the cohesiveness of the trophectoderm (A–C; A being the highest). In this study, blastocysts were divided into 4 grades based on their morphological score: grade 1, the blastocyst score was 4AA; grade 2, the blastocyst score was 4AB or 4BA; grade 3, the blastocyst score was 4BB; and grade 4, the blastocyst score was 4BC. In addition, blastocyst with score from grade 1 to 3 was defined as top-quality (TQ) blastocyst.

### SBCM Sample Collection

After blastocysts were frozen, 30 μl SBCM were collected and stored at −80°C until further analysis. Only the SBCMs from the blastocysts which developed on D5 and from top-quality cleavage embryos formed by the fertilized zygote of 2PN were collected. The SBCM without blastocysts under the same condition were collected as negative control (NC) samples (NCs) (n = 6). Furthermore, samples of Quinn’s Advantage Blastocyst Medium without serum protein substitute were also collected (n = 6).

### GM-CSF Detection by SIMOA Platform

The concentration of GM-CSF in SBCMs was measured using a GM-CSF assay kit (Cat No: 102329) following the manufacturer’s protocol (Quanterix, Billerica, MA, USA) on an HD-1 platform. SBCMs were diluted at 1:4 ratio and were performed in singlicate. The limit of detection (LOD) of GM-CSF is 0.50 fg/ml. SBCMs with GM-CSF concentration lower than LOD were assigned 0.50 fg/ml.

### Statistical Analysis

Statistical analysis was performed using SPSS version 23 (IBM company, Chicago, IL, USA). Data were expressed as mean ± standard deviation (SD) for variables with a normal distribution. Data were presented as median (25th and 75th percentile) for variables with a non-normal distribution. Categorical variables were expressed in ratios and quantities. Statistical differences between groups were determined using the ANOVA test for quantitative data, chi-square test for comparing frequencies. The correlation values between the qualitative datas were calculated by Spearman’s rank correlation coefficient test. To determine the predictive value of GM-CSF for pregnancy outcome, receiver operating characteristic (ROC) curve analyses were performed. The predicted probability of logistic regression model being diagnosed with pregnancy outcome was used as a surrogate marker to construct ROC curve (17). The area under the curve (AUC) was used as an accuracy index for evaluating the predictive values of GM-CSF for predicting pregnancy outcomes. A *p*-value of <0.05 was considered statistically significant.

## Results

### GM-CSF Concentration in SBCM as Detected by SIMOA

A total of 126 SBCMs were tested, and GM-CSF was detected in 63 SBCMs of them, and the detection rate was 50%. The concentration of GM-CSF in the detected SBCMs was 5.60 (1.95, 14.74) fg/ml. GM-CSF was detected in all the NCs and not in the Quinn’s Advantage Blastocyst Medium samples without serum protein substitute. The concentration of GM-CSF in NCs was 3.72 (3.58, 4.05) fg/ml (**Table 1**).

**Table 1:** GM-CSF concentration in SBCM detected by SIMOA.

	Detection rate (%)	Concentration (fg/mL)^a^
SBCM	50 (63/126)	5.60 (1.95, 14.74)
NC	100 (6/6)	3.72 (3.58, 4.05)
Quinn’s Advantage Blastocyst Medium	0 (0/6)	<0.50

^a^The concentration of GM-CSF in the detected samples was presented using median (25th, 75th percentile); SBCM, single-blastocyst conditioned medium; NC, negative control.

### Relationship Between Levels of GM-CSF in SBCM and Embryo Quality

To investigate the relationship of GM-CSF and embryo quality, the rate of TQ blastocyst was compared among the groups with different concentration of GM-CSF. Based on the median concentration of GM-CSF in NCs (3.72 fg/ml) and the LOD (0.50 fg/ml), all the 126 blastocysts were divided into three groups: high concentration group (HIGH, >3.72 fg/ml), mid concentration group (MID, ≤3.72 & >0.50 fg/ml), and low concentration group (LOW, ≤0.50 fg/ml). As presented in **Table 2**, there was no significant difference in the age of the patients, body mass index (BMI), infertility duration, primary infertility rate, and the number of retrieved oocytes among the three groups. The TQ blastocyst rate in the HIGH group, MID group, and the LOW group was 80.00% (28/35), 53.57% (15/28), and 49.21% (31/63). There was significant difference in the TQ blastocyst rate among the three groups (*p* = 0.010). The correlation values between GM-CSF concentration group and morphological score (MS) was 0.245 (*p* = 0.006).

**Table 2:** Relationship of GM-CSF concentration in SBCM with embryo quality.

	HIGH	MID	LOW	*p*
**Patients’ characteristics**				
n	35	28	63	
Female age (years)	32.09 ± 3.92	31.46 ± 3.08	30.78 ± 3.19	0.164
BMI (kg/m2)	22.19 ± 3.13	21.01 ± 2.29	21.58 ± 2.76	0.246
Infertility duration (years)	2.71 ± 1.35	2.93 ± 2.00	3.41 ± 2.49	0.527
Primary infertility, n (%)	42.86 (15)	42.86 (12)	50.79 (32)	0.671
No. of retrieved oocytes	13.66 ± 3.11	13.89 ± 3.58	13.96 ± 3.77	0.948
**Outcomes**				
Top-quality embryo rate, n (%)	80.00 (28)	53.57 (15)	49.21 (31)	0.010*

Data with a normal distribution were expressed as mean ± standard deviation (SD) and for variables Categorical variables were expressed in ratios and quantities. HIGH, high concentration group; MID, mid concentration group; LOW, low concentration group; BMI, body mass index. p < 0.05 was considered statistically significant. *p < 0.05.

### Relationship Between Levels of GM-CSF in SBCM and Pregnancy Outcome

To further investigate the relationship of GM-CSF and embryo developmental potential, the rates of HP, CP and OP were compared among the groups with different concentration of GM-CSF, respectively. All the 77 blastocysts, which were transferred into maternal uterus, were also divided into three groups based on GM-CSF concentration. As presented in **Table 3**, there was no significant difference in the age of patients, BMI, infertility duration, primary infertility rate, number of retrieved oocytes, and endometrial thickness on the day of hCG administration among the three groups. The HP rate in the HIGH group was significantly higher (86.67%) than that in the MID (66.67%) and LOW (55.17%) group (*p* = 0.029). The CP rate in the HIGH group was significantly higher (80.00%) than that in the MID (50.00%) and LOW (34.48%) group (*p* = 0.002). The OP rate in the HIGH group was also significantly higher (73.33%) than that in the MID (38.89%) and LOW (20.69%) group (p <0.001). The correlation values between GM-CSF concentration group and HP, CP and OP was 0.302 (p=0.008), 0.402 (*p <*0.001), and 0.464 (*p <*0.001), respectively.

**Table 3:** Association of GM-CSF concentration in FET-SBCM with the pregnancy outcome.

	HIGH	MID	LOW	*p*
**Patients’ characteristics**				
n	30	18	29	
Female age (years)	32.43 ± 3.92	31.56 ± 3.11	30.97 ± 3.80	0.270
BMI (kg/m2)	22.44 ± 3.20	21.54 ± 2.22	21.38 ± 1.91	0.325
Infertility duration (years)	2.75 ± 1.37	2.22 ± 1.06	3.67 ± 2.91	0.245
Primary infertility, n (%)	43.33 (13)	50.00 (9)	44.83 (13)	0.901
No. of retrieved oocytes	13.70 ± 3.12	13.83 ± 3.14	13.17 ± 2.63	0.467
Endometrial thickness (mm)	9.30 ± 2.38	8.55 ± 2.50	9.10 ± 0.90	0.321
**Outcomes**				
HCG positive rate, n (%)	86.67 (26)	66.67 (12)	55.17 (16)	0.029*
Clinical pregnancy rate, n (%)	80.00 (24)	50.00 (9)	34.48 (10)	0.002**
Ongoing pregnancy rate, n (%)	73.33 (22)	38.89 (7)	20.69 (6)	<0.001***


Data with a normal distribution were expressed as mean ± standard deviation (SD) and for variables Categorical variables were expressed in ratios and quantities. HIGH, high concentration group; MID, mid concentration group; LOW, low concentration group; BMI, body mass index. p < 0.05 was considered statistically significant. *p < 0.05; **p < 0.010; ***p < 0.001.

### The Predictive Value of GM-CSF for Pregnancy Outcome

**Figure 1:**
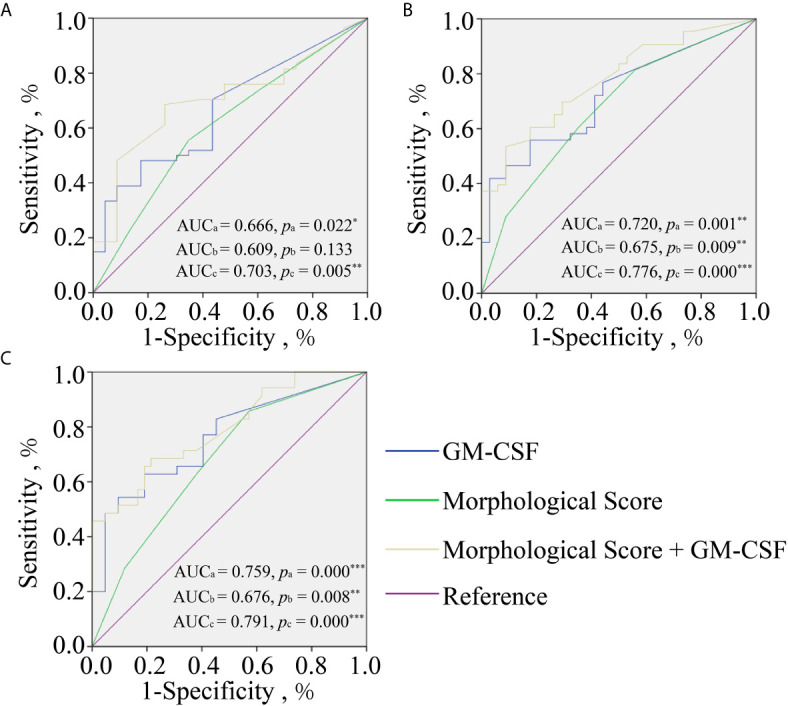
Receiver operating characteristic curve analyses of GM-CSF alone (Blue line), morphological score (MS) alone (green line), and morphological score combined with GM-CSF (yellow line) for predicting pregnancy outcomes at different gestation periods including HCG positive **(A)**; clinical pregnancy **(B)**; ongoing pregnancy **(C)**. AUC_a_ and *P*_a_ for GM-CSF, AUC_b_ and *P*_b_ for MS, and AUC_c_ and *P*_c_ for combined indicator. ^*^ *p* < 0.05; ^**^ *p* < 0.010; ^***^ *p* < 0.001.

To further analyze the power of GM-CSF in predicting pregnancy outcomes, ROC curve analyses of MS, GM-CSF, and combined indicator with MS and GM-CSF (Combination) for predicting HP, CP, and OP were performed, respectively (**Figure 1**). The logit model used to draw the ROC curve is presented in **Table 4**. For HP prediction (**Figure 1A** and **Table 4**), the AUC of three indicators (MS, GM-CSF, and Combination) were 0.609 (95% CI: 0.472–0.746; Cutoff = 0.704; Sensitivity = 0.556; Specificity = 0.652), 0.666 (95% CI: 0.540–0.792; Cutoff = 0.687; Sensitivity = 0.481; Specificity = 0.826) and 0.703 (95% CI: 0.583–0.823; Cutoff = 0.682; Sensitivity = 0.685; Specificity = 0.739), respectively. For CP prediction (**Figure 1B** and **Table 4**), the AUC of three indicators were 0.675 (95% CI: 0.555–0.796; Cutoff = 0.455; Sensitivity = 0.814; Specificity = 0.674), 0.720 (95% CI: 0.608–0.833; Cutoff = 0.529; Sensitivity = 0.558; Specificity = 0.824) and 0.776 (95% CI: 0.674–0.878; Cutoff = 0.666; Sensitivity = 0.535; Specificity = 0.912), respectively. For OP prediction (**Figure 1C** and **Table 4**), the AUC of three indicators were 0.676 (95% CI: 0.556–0.796; Cutoff = 0.359; Sensitivity = 0.857; Specificity = 0.429), 0.759 (95% CI: 0.650–0.869; Cutoff = 0.529; Sensitivity = 0.543; Specificity = 0.905) and 0.791 (95% CI: 0.691–0.892; Cutoff = 0.456; Sensitivity = 0.686; Specificity = 0.786), respectively. To predict different pregnancy outcomes, the ROC curve analyses revealed that Combination panels had the highest AUC, while the AUC of GM-CSF panels was larger than that of MS groups.

**Table 4:** Receiver operating characteristic curve analyses among different groups.

		Logit model	AUC	Cutoff	Sensitivity	Specificity	95% CI		*p*
							Lower	Upper	
HP	GM-CSF	LogP = 106.461C + 0.346	0.666	0.687	0.481	0.826	0.540	0.792	0.022*
	MS	LogP = 0.944M1 + 0.069M2 + 0.839M3 + 0.442	0.609	0.704	0.556	0.652	0.472	0.746	0.133
	Combination	LogP = 0.689M1 – 0.139M2 + 0.815M3 + 113.384C + 0.014	0.703	0.682	0.685	0.739	0.583	0.823	0.005**
CP	GM-CSF	LogP = 145.234C – 0.486	0.720	0.529	0.558	0.824	0.608	0.833	0.001**
	MS	LogP = 2.015M1 + 0.880M2 + 1.070M3 – 0.629	0.675	0.455	0.814	0.674	0.555	0.796	0.009**
	Combination	LogP = 1.961M1 + 0.759M2 + 1.165M3 + 148.263C – 1.350	0.776	0.666	0.535	0.912	0.674	0.878	<0.001***
OP	GM-CSF	LogP = 154.695C – 1.039	0.759	0.529	0.543	0.905	0.650	0.869	<0.001***
	MS	LogP = 1.974M1 + 1.281M2 + 1.368M3 – 1.281	0.676	0.359	0.857	0.429	0.556	0.796	0.008**
	Combination	LogP = 2.038M1 + 1.358M2 + 1.663M3 + 159.516C – 2.300	0.791	0.456	0.686	0.786	0.691	0.892	<0.001***

Note: HP, HCG positive; CP, clinical pregnancy; OP, ongoing pregnancy; MS, morphological score; Combination, morphological score combined with GM-CSF. C, Concentration of GM-CSF; M1, Morphological score was grade1; M2, Morphological score was grade2; M3, Morphological score was grade 3. p < 0.05 was considered statistically significant. *p < 0.05; **p < 0.010; ***p < 0.001.

## Discussion

The selection of embryos with the highest implantation potential for transfer is the key to successful pregnancy (18). To the best of our knowledge, the present study for the first time quantitatively detected the concentration of GM-CSF in SBCM and revealed that the concentration of GM-CSF in SBCM was positively associated with the embryo quality and pregnancy outcome. More importantly, results from the ROC analysis revealed that GM-CSF concentration exhibited a good predictive value for pregnancy outcomes. Taken together, these results indicated that GM-CSF could be used as a biomarker for the noninvasive selection of embryos with the highest developmental potential for transfer in ART.

Although the expression and secretion of GM-CSF in preimplantation embryos have been well-recognized (5, 10), the association of concentration of GM-CSF with the embryo quality and pregnancy outcome remains to be elucidated. Recently, two research groups have tried to detect GM-CSF in ECM using Luminex with LOD of 1.2 pg/ml as the detection method (19, 20). However, they could not detect the concentration of GM-CSF in the ECM, suggesting that the concentration of GM-CSF in the ECM may be in the fg/ml range, and therefore, too low to be measurable with Luminex. Previously, we found that SIMOA, a new ultrasensitive protein detection technology, can be used to determine proteins in SBCM at femtomolar concentrations (21). In the present study, the detection rate of GM-CSF in SBCMs was identified to be 50% (63/126), and the concentration of GM-CSF in the detected SBCM samples was 5.60 (1.95, 14.74) fg/ml. GM-CSF was also detected in all NCs at a concentration of 3.72 (3.58, 4.05) fg/ml. However, GM-CSF was not detected in medium without serum protein substitute, indicating that GM-CSF in NCs was derived from serum protein substitute.

An increasing number of studies have suggested that the addition of GM-CSF into the culture medium improves embryo quality in both human and mouse embryos (22, 23). It was demonstrated that GM-CSF receptors are expressed on the surface of embryo in humans (5), which could support the finding that GM-CSF can enhance cell survival and prevent apoptosis in freeze-thawed embryos (24). In the present study, in 35 of the 126 SBCMs tested, the concentration of GM-CSF was higher than that in NCs, indicating that the embryos cultured *in vitro* could secrete GM-CSF during development, besides, the concentration of GM-CSF in SBCM was positively correlated with the embryo quality. Therefore, we hypothesized that preimplantation embryos could promote their development through GM-CSF in an autocrine manner. Mechanistically, GM-CSF may function as a survival factor in embryos by promoting glucose uptake in embryos and providing protection from the stress response pathway and apoptosis through up-regulation of the expression of anti-apoptotic factor Bcl-2 (25). In addition, the concentration of GM-CSF in 91 of the 126 SBCMs was lower than that in the NCs. The potential reason for this result is that there might be a balance of secretion and consumption of GM-CSM. If the ability of GM-CSF secretion by the embryo was higher than that of its consumed during the embryonic development, the presence of GM-CSF was detectable in SBCMs, and vice versa. The ability of GM-CSF secretion and consumption seemed different in each individual embryo, which may indicate differences in embryo quality and its developmental potential.

The present study indicated that GM-CSF concentration was positively associated with the rate of HP, CP, and OP. Conversely, Dominguez et al. found that the concentration of GM-CSF in ECM of the implant group was lower than that of the non-implant group (12). The inconsistent findings between these two studies may be attributed to different experimental designs. Study conducted by Dominguez et al. was based on comparative quantitative detection of GM-CSF in multiple condition mediums mixtures, whereas this study was based on quantitative detection of GM-CSF in SBCMs. Moreover, our study revealed a wide range of GM-CSF concentration in different SBCMs, suggesting heterogeneity in the ability of embryos to secrete and consume GM-CSF. GM-CSF concentration in SBCM may better reflect the developmental potential of individual blastocysts.

To verify whether GM-CSF in SBCMs could be used as a biomarker for the selection of embryos for transplantation, we analyzed the predictive value of GM-CSF concentration in predicting pregnancy outcomes based on the ROC curve analyses. The AUC of GM-CSF in predicting pregnancy outcome was higher than MS. In addition, the predictive power of combination indicator with GM-CSF and MS in predicting pregnancy outcome was higher than single GM-CSF or MS. These findings indicated that GM-CSF exhibit a high predictive value for pregnancy outcome. Combined with the concentration of GM-CSF in SBCM, the accuracy of conventional MS in predicting pregnancy outcomes can be significantly improved. Taken together, these findings suggested that GM-CSF can be used as a promising biomarker for the selection of embryos with high developmental potential for transplantation.

Physiologically, GM-CSF is involved in at least two crucial processes during pregnancy, including the establishment and maintenance of an immune environment during pregnancy (26) and regulation of placental morphogenesis and function (27). Furthermore, Robertson et al. suggested that GM-CSF-deficiency leads to reduced fertility of mice (28). All these shreds of evidence suggested that the above-mentioned results are rational. Moreover, the study by Robertson et al. revealed that GM-CSF-deficient mice had a normal number of implantation sites in early pregnancy and the effect of GM-CSF-deficiency on pregnancy occurred mainly after implantation (28).

This study presents some limitations. First, the sample size in this study is relatively small. Thus, further studies with multicenter larger sample size are warranted to obtain a more accurate prediction model for clinical application. Second, it has been reported that embryo-secreted proteins, such as HCG (6, 29), soluble form of HLA-G (sHLA-G) (30, 31), and IL-6 (19, 21) also could be used to predict embryo quality or pregnancy outcome. The predictive model based on multiple proteins in SBCMs may improve the sensitivity and specificity of the prediction model in the further study. Nevertheless, this study provides a basis for further research.

In conclusion, GM-CSF concentration in SBCM was determined for the first time by a quantitative assay in this study. Furthermore, we also identified that GM-CSF concentration in SBCM was positively associated with embryo quality and pregnancy outcome. In addition, single GM-CSF or combined with MS exhibited good predictive value for pregnancy outcome. All these data indicated that GM-CSF might serve as a biomarker to select embryos with high developmental potential to achieve successful pregnancy.

## Data Availability Statement

The original contributions presented in the study are included in the article/supplementary material. Further inquiries can be directed to the corresponding authors.

## Ethics Statement

The studies involving human participants were reviewed and approved by the Research Ethics Committee of Shenzhen Zhongshan Urology Hospital (Approval number: SZZSECHU-20180021). Written informed consent for participation was not required for this study in accordance with the national legislation and the institutional requirements.

## Author Contributions

YZ and LD were involved in study design. PC and CH were involved in the organization of the entire project, date analysis and manuscript writing. QS, HZ, FX, ZY, and CW were involved in embryo culture, formulation of clinical sample collection scheme and sample collection. SL and ZL were mainly involved in date analysis. All authors contributed to the article and approved the submitted version.

## Funding

This study was funded by the National Key Research & Developmental Program of China (2018YFC1003900/2018YFC1003904), clinical research special fund of Chinese Medical Association (18010120741), Shenzhen Natural Science Foundation (JCYJ20190813161801676), Basic Research Program of Shenzhen (JCYJ20160427113153295), National Natural Science Foundation of China (21807072) and Sanming Project of Medicine in Shenzhen (SZSM201502035).

## Conflict of Interest

The authors declare that the research was conducted in the absence of any commercial or financial relationships that could be construed as a potential conflict of interest.

## Abbreviations

MS, Morphological score; Combination, Morphological score combined with GM-CSF; HP, HCG positive; CP, Clinical pregnancy; OP, Ongoing pregnancy; HIGH, High concentration group; MID, Mid concentration group; LOW, Low concentration group; SBCM, Single-blastocyst conditioned medium; NC, Negative control.
